# Morphology-defined bacterial vaginosis and HPV-related cervical screening abnormalities: a two-year real-world study with histopathologic correlation

**DOI:** 10.3389/fcimb.2026.1890650

**Published:** 2026-07-22

**Authors:** Zhen Li, Zhi Duan, Ran Liu, Siwei Yu, Ning Wang, Hui Chen, Qimei Xu

**Affiliations:** 1 Department of Pathology, The First Hospital of Changsha/The Affiliated Changsha Hospital of Xiangya School of Medicine, Central South University, Changsha, Hunan, China; 2 Changsha High-Throughput Sequencing Technology Innovation Center for Gastrointestinal Tumor Precision Medicine, Changsha, Hunan, China; 3 Department of Infectious and Immunology, The First Hospital of Changsha/The Affiliated Changsha Hospital of Xiangya School of Medicine, Central South University, Changsha, Hunan, China

**Keywords:** bacterial vaginosis, cervical cytology, cervical histopathology, human papillomavirus, vaginal microecology

## Abstract

**Background:**

Bacterial vaginosis (BV) is characterized by reduced Lactobacillus dominance and enrichment of anaerobic bacteria. Although BV has been associated with human papillomavirus (HPV) infection, its relationship with cytologic abnormalities and biopsy-confirmed cervical lesions remains incompletely defined in real-world laboratory settings.

**Methods:**

We conducted a retrospective real-world study integrating vaginal fluorescence microscopy, 21-genotype HPV genotyping, thin-prep cytology (TCT), and cervical histopathology records from January 2024 through December 2025 at a tertiary hospital in China. Morphology-defined BV was defined by clue cells or Gardnerella-like anaerobic bacteria on vaginal fluorescence microscopy and was not equivalent to Nugent scoring, Amsel criteria, culture-based diagnosis, or molecular microbiome profiling. HPV and TCT records were matched within a prespecified 30-day window after patient-level deduplication. Multivariable logistic regression adjusted for year, age, and vaginal microecological covariates. A 90-day sensitivity analysis was performed. The histopathology cohort was clinically selected and was analyzed as a correlation subgroup rather than as a random sample of the screening cohort.

**Results:**

The main 30-day analysis included 4,492 unique patients, of whom 587 (13.07%) had morphology-defined BV. After multivariable adjustment, morphology-defined BV remained associated with overall HPV positivity (adjusted odds ratio [aOR] 1.560; 95% confidence interval [CI] 1.293–1.883), high-risk HPV positivity (aOR 1.611; 95% CI 1.327–1.957), HPV multiple infection (aOR 1.976; 95% CI 1.526–2.559), TCT abnormality (aOR 1.465; 95% CI 1.125–1.907), and concurrent high-risk HPV positivity plus TCT abnormality (aOR 1.558; 95% CI 1.174–2.066). After additional adjustment for high-risk HPV, the BV–TCT association was attenuated and non-significant. Among 825 patients with cervical histopathology records, BV was not independently associated with any histologic lesion grade, whereas high-risk HPV, HPV16/18, and TCT abnormality were the principal predictors of histopathologic disease.

**Conclusion:**

Morphology-defined BV was associated with HPV infection and HPV-related cytologic abnormalities, but not with histologic cervical lesions in the clinically selected biopsy subgroup. These findings suggest that routine morphologic evidence of BV marks an HPV-related screening-positive phenotype rather than an independent histopathologic lesion predictor.

## 1 Introduction

Cervical cancer remains a major global health burden despite being largely preventable through HPV vaccination, screening, and treatment of precancerous lesions (Arbyn et al., 2020; Sung et al., 2021). Persistent infection with high-risk HPV is the necessary causal factor for most cervical cancers and their precursor lesions (Walboomers et al., 1999; Wallin et al., 1999). However, HPV infection alone does not fully explain the heterogeneity of cervical disease progression. Most HPV infections are transient, and only a fraction progress to clinically significant intraepithelial lesions or invasive cancer. Viral genotype, host immunity, local inflammation, co-infections, and the vaginal microenvironment may influence HPV acquisition, persistence, and progression (Cui et al., 2025).

The vaginal microbiota has attracted increasing attention as a modifiable local factor related to HPV infection and cervical carcinogenesis. A Lactobacillus-dominant vaginal ecosystem is generally considered protective, whereas BV is characterized by reduced lactobacilli, enrichment of anaerobic bacteria, increased vaginal pH, epithelial barrier disruption, and mucosal inflammation (Norenhag et al., 2020; Wu et al., 2022; Zukiene et al., 2025). Standard BV diagnostic approaches include clinical Amsel criteria, Gram-stain-based Nugent scoring, and molecular diagnostic methods (Nugent et al., 1991; Savicheva, 2024). Meta-analytic evidence supports positive associations between BV and cervical HPV infection, although the effect estimates and certainty vary across diagnostic methods and populations (Martins et al., 2022). In routine pathology and microbiology laboratories, vaginal microecology is also assessed by morphology-based microscopy, which can identify clue cells, Gardnerella-like anaerobic bacteria, fungal elements, inflammatory cells, lactobacilli, cleanliness grade, and pH-related abnormalities as part of the diagnostic workflow.

Less is known about where morphology-defined BV fits within the combined pathology-microbiology pathway that links vaginal microecology, HPV genotyping, cervical cytology, and histopathologic lesion assessment. This distinction is important for laboratory interpretation. If BV is associated mainly with HPV positivity and cytologic abnormalities but not with biopsy-confirmed lesions, it may represent an infection-related or inflammatory screening phenotype rather than an independent marker of established cervical tissue disease (Xu et al., 2022).

Vaginal fluorescence microscopy is used in routine gynecologic laboratory practice in China to evaluate vaginal microecological morphology. It identifies clue cells, Gardnerella-like anaerobic bacteria, Candida-like fungal elements, leukocytes, lactobacilli, cleanliness grade, and other morphologic findings under fluorescence microscopy. This approach is practical and scalable, but it is not equivalent to established BV diagnostic standards such as Amsel criteria, Nugent scoring, culture-based diagnosis, or molecular microbiome profiling. Therefore, the exposure in this study should be interpreted strictly as morphology-defined BV. Differences in sensitivity, specificity, and diagnostic thresholds may limit direct comparability with studies using Nugent, Amsel, or sequencing-based definitions (Feng et al., 2023).

In the present study, we integrated vaginal fluorescence microscopy, HPV genotyping, TCT cytology, and cervical histopathology data from January 1, 2024, through December 31, 2025. Using patient-level deduplication, prespecified temporal matching windows, multivariable logistic regression, and sensitivity analyses, we evaluated whether morphology-defined BV was associated with HPV infection patterns, cytologic abnormality, combined HPV-cytology abnormalities, and histologic cervical lesions in a two-year real-world pathology-microbiology dataset.

## 2 Materials and methods

### 2.1 Study design and data source

This was a retrospective real-world pathology-microbiology study based on routinely collected clinical laboratory and pathology data from Changsha First Hospital, a tertiary hospital in Changsha, Hunan, China.

### 2.2 Eligibility criteria

Eligible patients were women who underwent vaginal fluorescence microscopy because of clinically suspected vaginitis or other vaginal infection-related conditions, including bacterial vaginosis and vulvovaginal candidiasis. Eligible specimens were vaginal or introitus secretions that met routine clinical specimen quality requirements and were adequate for immunofluorescence-based morphologic assessment.

Patients were excluded if essential clinical information was incomplete, if the main clinical condition was a non-vaginal upper genital tract infection such as endometritis or salpingitis rather than vaginal infection, or if the specimen was improperly stored or otherwise unsuitable for fluorescence microscopy. Pregnancy status, HPV vaccination status, sexual behavior, contraceptive use, smoking, prior cervical procedures, and recent antimicrobial exposure were not consistently available in the routine data system and therefore could not be used as exclusion criteria or covariates.

### 2.3 Specimen collection

For patients with a history of sexual activity, vaginal secretions were collected from the posterior vaginal fornix using a sterile cotton swab. For patients without a history of sexual activity, secretions were collected from the vaginal introitus using a sterile cotton swab. After collection, the swab was placed into a clean specimen tube containing a small amount of normal saline at the bottom and immediately transported to the Department of Pathology for vaginal secretion immunofluorescence testing.

### 2.4 Vaginal fluorescence microscopy

Vaginal secretion material was evenly smeared onto two glass slides. One slide was treated with solution A and the other with solution B from immunochromogenic reagent III. After coverslip placement, slides were examined using a biological fluorescence microscope equipped with ultraviolet and/or B-band fluorescence modules. Morphologic interpretation was performed at 20×, 40×, and 100× magnification by attending-level or higher physicians in the Department of Pathology with experience in cytologic and histologic diagnosis.

The instrument was an Olympus BX53 fluorescence microscope with an excitation wavelength range of 330–380 nm. Reagents were supplied by Shanghai Kaijing Biotechnology Co., Ltd. BV positivity was defined as the presence of clue cells or Gardnerella-like anaerobic bacteria on vaginal fluorescence microscopy. This definition was treated as a routine laboratory morphology-defined exposure rather than as a Nugent, Amsel, culture-based, or molecular microbiome diagnosis. Other vaginal microecological variables included cleanliness grade, lactobacillus reduction or absence, leukocytes or pus cells ≥16 per high-power field, vulvovaginal candidiasis morphology, other bacterial morphology, and vaginal pH. No local validation study comparing this fluorescence microscopy definition with Nugent scoring or Amsel criteria was available. Formal interobserver agreement for Gardnerella-like anaerobic bacteria or clue-cell identification was not assessed in this retrospective dataset. Therefore, potential exposure misclassification and limited comparability with studies using standard BV diagnostic criteria were considered when interpreting all BV-related analyses.

### 2.5 HPV genotyping

HPV genotyping was performed using the 21-Genotype HPV Genotyping Kit (Hybribio Co., Ltd., Guangzhou, China) based on a three-step PCR amplification–flow-through hybridization–low-density gene chip method. Cervical specimens were processed by alkaline lysis (100 °C, 15 minutes) followed by high-speed centrifugation (14,000 rpm, 5 minutes) for DNA extraction. Extracted DNA was amplified by PCR using biotin-labeled consensus primers targeting the conserved L1 region of HPV (pre-denaturation at 95 °C for 5 minutes; 40 cycles of 95 °C for 30 seconds, 55 °C for 30 seconds, and 72 °C for 45 seconds; final extension at 72 °C for 5 minutes). Amplicons were hybridized on a low-density gene chip carrying type-specific oligonucleotide probes for 21 HPV genotypes using the HybriMax^®^ nucleic acid hybridization system (Hybribio Co., Ltd.), and hybridization dot patterns were interpreted according to the chip reference map by trained laboratory personnel. Each run included a positive control and a negative control; an internal amplification control (IC) and a biotin hybridization control verified run validity, and results from runs with invalid controls were excluded.

The kit detects 15 high-risk genotypes (HPV 16, 18, 31, 33, 35, 39, 45, 51, 52, 53, 56, 58, 59, 66, and 68) and 6 low-risk genotypes (HPV 6, 11, 42, 43, 44, and 81), consistent with expanded high-risk classifications used in Chinese clinical practice guidelines; HPV 53 and 66 are classified as possibly carcinogenic (Group 2B) by the International Agency for Research on Cancer. HPV-related outcomes included overall HPV positivity, high-risk HPV positivity (one or more of the 15 high-risk genotypes), HPV16/18 positivity (HPV16 and/or HPV18), and HPV multiple infection (two or more genotypes of any risk category). Low-risk HPV positivity was summarized descriptively but was not included as a main multivariable endpoint. HPV persistence, viral clearance, and viral load were not evaluable because repeat longitudinal HPV testing and quantitative measurements were unavailable.

### 2.6 Thin-prep cytology test procedure

Cervical cytology specimens were collected by the attending gynecologist using a cervical sampling brush, placed immediately into liquid-based cytology preservative solution, and processed using a sedimentation-based liquid-based cytology system (LBP System; Guangzhou Anbiping Medical Technology Co., Ltd., Guangzhou, China). Briefly, each specimen was vortex-mixed (3,000 rpm, 15–20 seconds) and transferred into a centrifuge tube containing 4 mL density-gradient separation medium. Sequential centrifugation was performed: a first low-speed spin (200 × g, 2 minutes) to remove erythrocytes and mucus, followed by aspiration of the supernatant, and a second high-speed spin (800 × g, 5 minutes) to concentrate the cellular pellet. The resuspended cell suspension was then processed by the automated slide preparation unit to deposit a uniform thin-cell monolayer onto a labeled glass slide. Slides were fixed in absolute ethanol and stained using a modified Papanicolaou protocol (hematoxylin, 85 seconds; EAOG counterstain, 180 seconds), followed by graded ethanol dehydration and turpentine clearing before coverslipping.

Initial cytologic interpretation was performed independently by two qualified pathologists—one attending-level physician and one physician holding a senior professional title—who were blinded to both vaginal fluorescence microscopy and HPV results. For cases with abnormal TCT findings, HPV results could be reviewed during post-interpretation clinical correlation, but HPV information was not used to change the cytologic diagnosis. Discrepant cytologic interpretations were resolved by consensus. Results were reported according to the 2014 Bethesda System. Cytologic abnormality was defined as atypical squamous cells of undetermined significance or worse (ASC-US+).

### 2.7 Cytology and histopathology outcomes

Additional cytologic severity outcomes included LSIL or worse. The main combined screening outcome was concurrent high-risk HPV positivity and TCT abnormality in the same matched record.

Histopathology was analyzed as a downstream clinical outcome among patients with matched cervical pathology records. Histopathology reports were reviewed using targeted text extraction focused on cervical or endocervical lesions. Target lesions included koilocytosis, low-grade squamous intraepithelial lesion (LSIL), high-grade squamous intraepithelial lesion (HSIL), squamous cell carcinoma, and adenocarcinoma. Koilocytosis alone was included under “any target lesion” but was not counted as LSIL or worse unless the pathology report explicitly diagnosed LSIL or a higher-grade lesion. Diagnoses attributable to endometrial carcinoma or non-cervical uterine cavity disease were excluded from cervical lesion classification. When multiple pathology records or multiple diagnostic terms were present for the same patient, the highest-grade cervical lesion was used. Histologic outcomes included any target lesion, LSIL or worse, HSIL or worse, and cancer. Because only 38 cancer cases were identified, cancer-specific models were interpreted as exploratory and descriptive.

### 2.8 Matching and deduplication

The vaginal fluorescence microscopy dataset was used as the base dataset. HPV, TCT, and histopathology records were matched first by patient identification number, then by patient hospital number or outpatient number when the identification number was unavailable. For HPV and TCT matching, the closest report date to the vaginal fluorescence report date was selected. The prespecified main matching window required both HPV and TCT results to be within 30 days of the vaginal fluorescence report. A 90-day matching window was used as sensitivity analysis. After matching, records were deduplicated at the patient level across 2024 and 2025, retaining one eligible record per patient according to predefined analytic rules.

The main 30-day screening analysis included 4,492 unique patients. The 90-day sensitivity analysis included 4,562 unique patients. The final histopathology analysis included 825 deduplicated patients with cervical pathology records.

### 2.9 Statistical analysis

Continuous variables were summarized as medians and interquartile ranges. Categorical variables were summarized as counts and percentages. Between-group comparisons used chi-square or Fisher exact tests for categorical variables and Mann-Whitney U tests for continuous variables, as appropriate. Univariable associations between BV and HPV-related outcomes, cytologic outcomes, and histopathologic outcomes were estimated using odds ratios (ORs) and 95% confidence intervals (CIs). The primary outcomes were overall HPV positivity, high-risk HPV positivity, and HPV multiple infection. Secondary outcomes were TCT abnormality and concurrent high-risk HPV positivity with TCT abnormality. Histopathologic outcomes were analyzed as downstream pathology correlations among patients with available cervical pathology records. Multivariable logistic regression models were used to estimate adjusted odds ratios (aORs).

For HPV-related and cytologic outcomes, models included BV as the primary exposure and adjusted for year, age, cleanliness grade III/IV, lactobacillus reduction or absence, leukocytes or pus cells ≥16 per high-power field, VVC morphology, and other bacterial morphology. Covariates were selected on the basis of clinical and laboratory relevance rather than automated P-value selection. The same adjustment strategy was used for TCT abnormality and the combined high-risk HPV plus TCT abnormality outcome. For cytologic abnormality, an additional model further adjusted for high-risk HPV positivity; this was interpreted as an attenuation analysis rather than a formal mediation analysis. For histopathologic outcomes, models evaluated BV together with established cervical disease markers, including high-risk HPV positivity, HPV16/18 positivity, HPV multiple infection, and TCT abnormality. Age was entered into logistic regression models as a continuous variable per 1-year increase. The linearity assumption for age was assessed descriptively by comparing models using age as a continuous variable with age-group summaries; because no major departure affecting the primary interpretation was observed, the continuous specification was retained for parsimony. To address potential overlap among HPV-related predictors in histopathology models, we performed a collinearity assessment using variance inflation factors (VIFs) for the full histopathology model. We also fitted alternative logistic regression models in which HR-HPV positivity, HPV16/18 positivity, HPV multiple infection, and TCT abnormality were entered separately or in reduced combinations. These analyses are reported in **Supplementary Tables S1**–**S3**.

The analyses were exploratory because multiple HPV-related, cytologic, and histopathologic endpoints were evaluated. Therefore, P values were interpreted with attention to consistency, effect size, and biological plausibility rather than as definitive confirmatory tests. Missing or unavailable routine clinical variables were not imputed; analyses used available matched records that met the prespecified eligibility and matching rules. Statistical analyses were performed using Python (version 3.11.9). All statistical tests were two-sided, and P values less than 0.05 were considered statistically significant.

## 3 Results

### 3.1 Cohort assembly and laboratory matching

**Figure 1:**
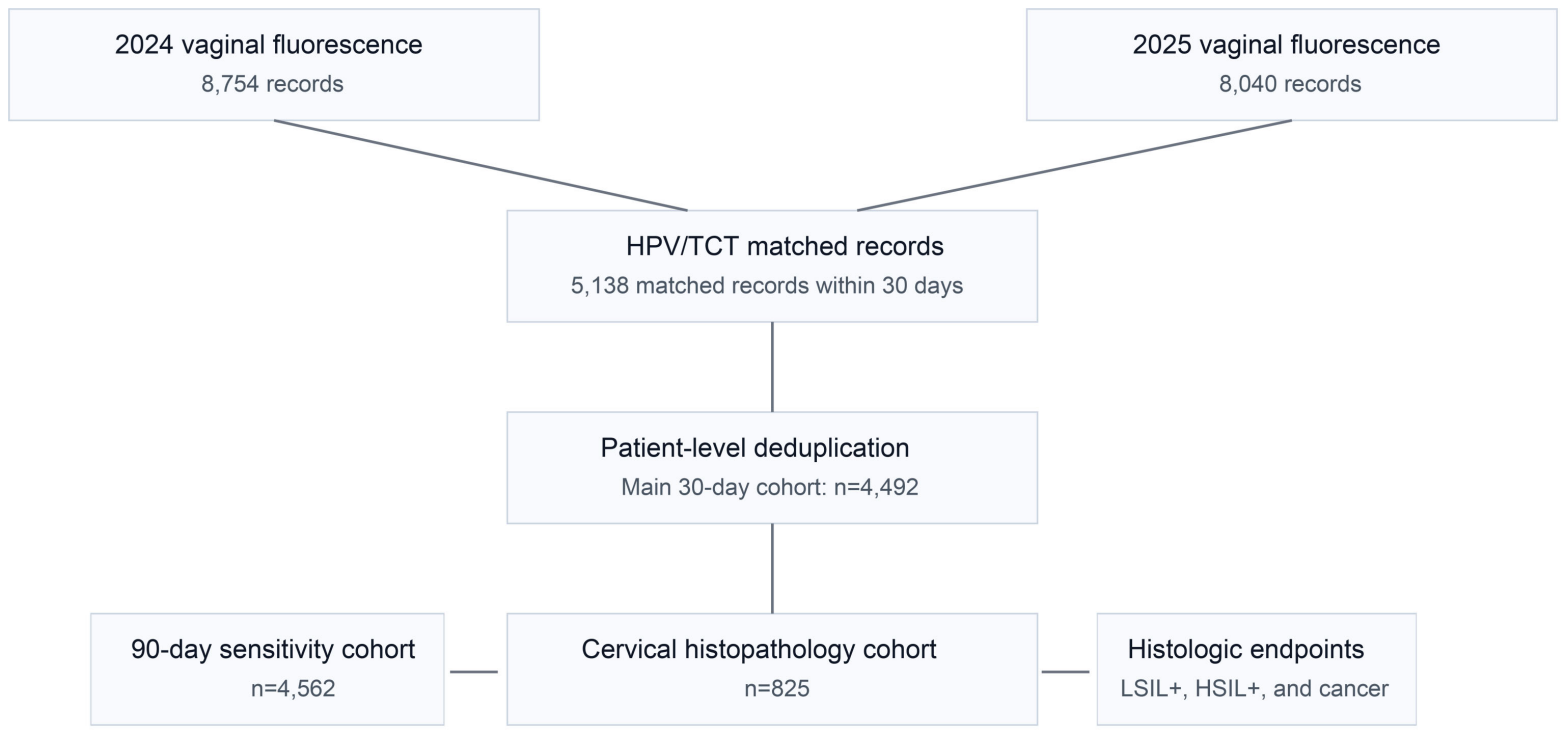
Study flow diagram. Flow diagram showing the source vaginal fluorescence microscopy records from 2024 and 2025, HPV/TCT matching within the 30-day main analysis window, patient-level deduplication, the 90-day sensitivity analysis cohort, the main screening cohort, and the cervical histopathology cohort.

After HPV/TCT matching and cross-year patient-level deduplication, the main 30-day analysis included 4,492 patients, including 2,382 from 2024 and 2,110 from 2025 (**Figure 1**). The median absolute time difference was 2 days for HPV testing and 3 days for TCT testing.

Morphology-defined BV was identified in 587 patients (13.07%). Overall HPV positivity, high-risk HPV positivity, HPV16/18 positivity, and HPV multiple infection occurred in 30.92%, 26.14%, 5.52%, and 10.28% of patients, respectively. TCT abnormality and concurrent high-risk HPV positivity plus TCT abnormality occurred in 10.86% and 9.06% of patients, respectively (**Tables 1**, **2**).

**Table 1:** Baseline characteristics of the 30-day main analysis cohort, stratified by BV status.

Characteristic	All patients (n=4,492)	BV positive (n=587)	BV negative (n=3,905)	P value
2025 study year	2,110 (46.97%)	207 (35.26%)	1,903 (48.73%)	<0.001
Age, median (IQR), years	38 (28-52)	40 (28-52)	38 (28-52)	0.678
Vaginal pH, median (IQR)	4.4 (3.80-5.10)	4.6 (4.40-5.10)	4.4 (3.80-5.10)	<0.001
Cleanliness grade III/IV	1,880 (41.85%)	388 (66.10%)	1,492 (38.21%)	<0.001
Reduced/absent lactobacilli	1,965 (43.74%)	382 (65.08%)	1,583 (40.54%)	<0.001
Leukocytes ≥16/HPF	2,472 (55.03%)	345 (58.77%)	2,127 (54.47%)	0.051
VVC morphology positive	896 (19.95%)	111 (18.91%)	785 (20.10%)	0.500
Other bacterial morphology positive	319 (7.10%)	38 (6.47%)	281 (7.20%)	0.525
Overall HPV positive	1,389 (30.92%)	231 (39.35%)	1,158 (29.65%)	<0.001
High-risk HPV positive	1,174 (26.14%)	202 (34.41%)	972 (24.89%)	<0.001
Low-risk HPV positive	428 (9.53%)	83 (14.14%)	345 (8.83%)	<0.001
HPV16/18 positive	248 (5.52%)	44 (7.50%)	204 (5.22%)	0.025
HPV multiple infection	462 (10.28%)	97 (16.52%)	365 (9.35%)	<0.001
TCT abnormality (ASC-US+)	488 (10.86%)	86 (14.65%)	402 (10.29%)	0.002

BV, bacterial vaginosis; HPV, human papillomavirus; TCT, thin-prep cytology test; ASC-US, atypical squamous cells of undetermined significance; IQR, interquartile range; VVC, vulvovaginal candidiasis.

**Table 2:** Association between morphology-defined BV and HPV-related laboratory outcomes.

Outcome	BV positive (n=587)	BV negative (n=3,905)	Univariable OR (95% CI)	Univariable P	Adjusted OR (95% CI)	Adjusted P
Overall HPV positivity	39.35%	29.65%	1.539 (1.287-1.841)	<0.001	1.560 (1.293-1.883)	<0.001
High-risk HPV positivity	34.41%	24.89%	1.583 (1.316-1.905)	<0.001	1.611 (1.327-1.957)	<0.001
HPV multiple infection	16.52%	9.35%	1.920 (1.506-2.448)	<0.001	1.976 (1.526-2.559)	<0.001
TCT abnormality (ASC-US+)	14.65%	10.29%	1.496 (1.164-1.923)	0.002	1.465 (1.125-1.907)	0.005
High-risk HPV positive + TCT abnormality	12.61%	8.53%	1.547 (1.183-2.024)	0.001	1.558 (1.174-2.066)	0.002

BV, bacterial vaginosis; HPV, human papillomavirus; TCT, thin-prep cytology test; ASC-US, atypical squamous cells of undetermined significance; OR, odds ratio; CI, confidence interval.

By year, BV positivity was lower in 2025 than in 2024 (9.81% vs. 15.95%), whereas HPV and cytology outcomes were similar between years. Overall HPV positivity was 30.56% in 2024 and 31.33% in 2025; high-risk HPV positivity was 25.90% and 26.40%, respectively; and TCT abnormality was 10.92% and 10.81%, respectively. This year-to-year difference in morphology-defined BV may reflect real changes in the tested clinical population, specimen collection, slide interpretation, or laboratory workflow. Because individual operator and process-level quality indicators were unavailable, year was included in multivariable models and year-specific descriptive results were retained for transparency.

Compared with BV-negative patients, BV-positive patients showed a less Lactobacillus-dominant vaginal microecological profile and higher rates of HPV-related screening abnormalities (**Table 1**).

### 3.2 Morphology-defined BV and HPV-related laboratory outcomes

**Figure 2:**
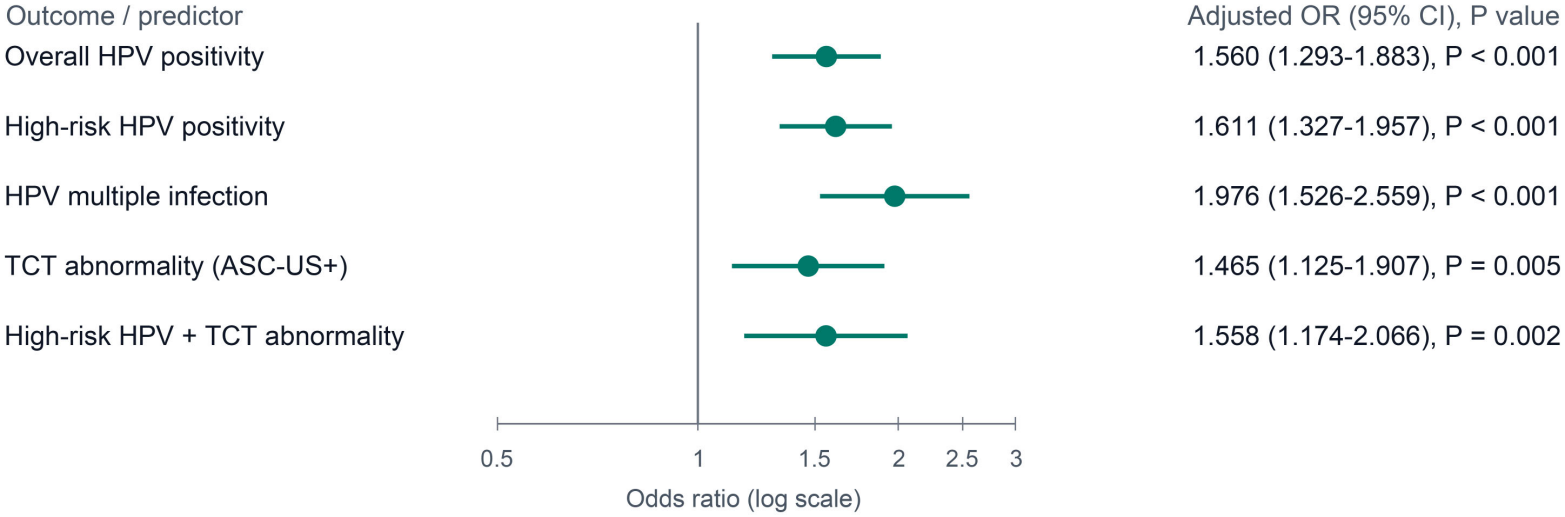
Association between morphology-defined BV and HPV-related laboratory outcomes. Forest plot showing adjusted odds ratios for the association of morphology-defined BV with overall HPV positivity, high-risk HPV positivity, HPV multiple infection, TCT abnormality, and concurrent high-risk HPV positivity plus TCT abnormality.

In univariable analysis, BV-positive patients had consistently higher rates of HPV infection outcomes than BV-negative patients, including overall HPV positivity, high-risk HPV positivity, and HPV multiple infection (**Table 2**; **Figure 2**).

After adjustment for year, age, and vaginal microecological factors, BV remained associated with overall HPV positivity (aOR 1.560; 95% CI 1.293–1.883), high-risk HPV positivity (aOR 1.611; 95% CI 1.327–1.957), and HPV multiple infection (aOR 1.976; 95% CI 1.526–2.559). The strongest association was observed for HPV multiple infection (**Table 2**).

### 3.3 HPV-related cytologic and combined screening outcomes

BV was also associated with HPV-related cytologic outcomes. After adjustment, BV remained associated with TCT abnormality (aOR 1.465; 95% CI 1.125–1.907) and concurrent high-risk HPV positivity plus TCT abnormality (aOR 1.558; 95% CI 1.174–2.066) (**Table 2**).

When TCT abnormality was additionally adjusted for high-risk HPV positivity, high-risk HPV remained the dominant predictor (aOR 21.128; 95% CI 16.416–27.193; P<0.001), whereas the association between BV and TCT abnormality was attenuated and no longer statistically significant (aOR 1.121; 95% CI 0.832–1.510; P = 0.454).

### 3.4 Temporal-window sensitivity analysis

The 90-day sensitivity analysis yielded results consistent with the main 30-day analysis. BV remained associated with all main HPV-related and screening outcomes, with adjusted ORs ranging from 1.455 for TCT abnormality to 1.966 for HPV multiple infection. Full temporal-window sensitivity results are provided in the **Supplementary Material**.

### 3.5 Histopathologic correlation

The histopathology analysis included 825 deduplicated patients with cervical pathology records. This subgroup represented a clinically selected higher-risk population: compared with the full 4,492-patient screening cohort, the histopathology subgroup had higher overall HPV positivity, high-risk HPV positivity, HPV16/18 positivity, and TCT abnormality, whereas BV positivity was similar or slightly lower. This enrichment indicates that biopsy referral was likely driven primarily by abnormal HPV and/or cytology results rather than by BV status.

**Figure 3:**
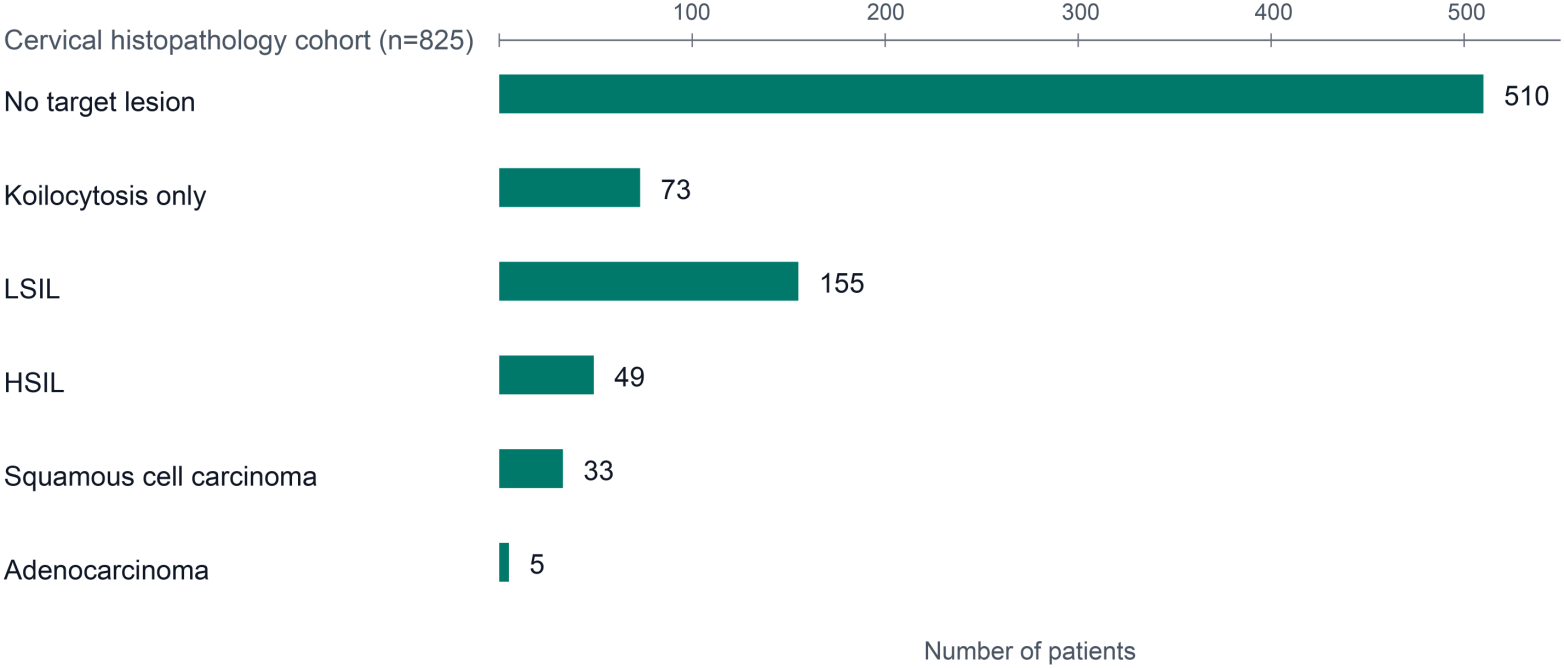
Distribution of highest histopathologic lesion categories. The bar chart shows mutually exclusive highest cervical histopathology categories among patients in the histopathology cohort (n=825).

Among the 825 patients, 315 (38.18%) had any target lesion, 242 (29.33%) had LSIL or worse, 87 (10.55%) had HSIL or worse, and 38 (4.61%) had cancer. Mutually exclusive highest lesion categories are summarized in **Figure 3**.

**Figure 4:**
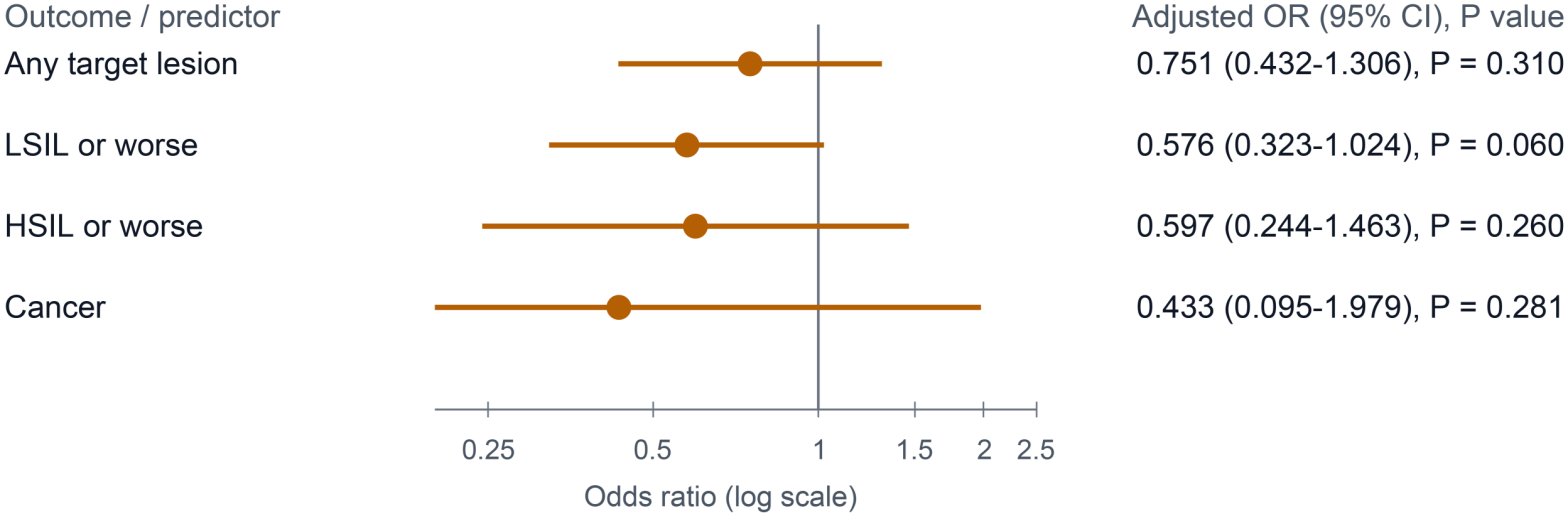
Association between BV and histopathologic cervical lesions. Forest plot showing adjusted odds ratios for the association of morphology-defined BV with any target lesion, LSIL or worse, HSIL or worse, and cancer in the cervical histopathology cohort.

BV was not significantly associated with histopathologic outcomes in either univariable or multivariable analyses, including any target lesion, LSIL or worse, HSIL or worse, and cancer (**Table 3** and **Figure 4**).

**Table 3:** Histopathologic outcomes and association with morphology-defined BV.

Histologic outcome	Positive cases, n (%)	Univariable OR for BV (95% CI)	Univariable P	Adjusted OR for BV (95% CI)	Adjusted P
Any target lesion	315 (38.18%)	1.079 (0.694-1.677)	0.735	0.751 (0.432-1.306)	0.310
LSIL or worse	242 (29.33%)	0.873 (0.537-1.417)	0.581	0.576 (0.323-1.024)	0.060
HSIL or worse	87 (10.55%)	0.663 (0.297-1.483)	0.314	0.597 (0.244-1.463)	0.260
Cancer	38 (4.61%)	0.425 (0.101-1.794)	0.230	0.433 (0.095-1.979)	0.281

BV, bacterial vaginosis; LSIL, low-grade squamous intraepithelial lesion; HSIL, high-grade squamous intraepithelial lesion; OR, odds ratio; CI, confidence interval. The histopathology cohort was clinically selected and should not be interpreted as a randomly sampled subset of the full screening cohort. BV was present in 93 of 825 patients (11.27%) in the histopathology cohort.

**Figure 5:**
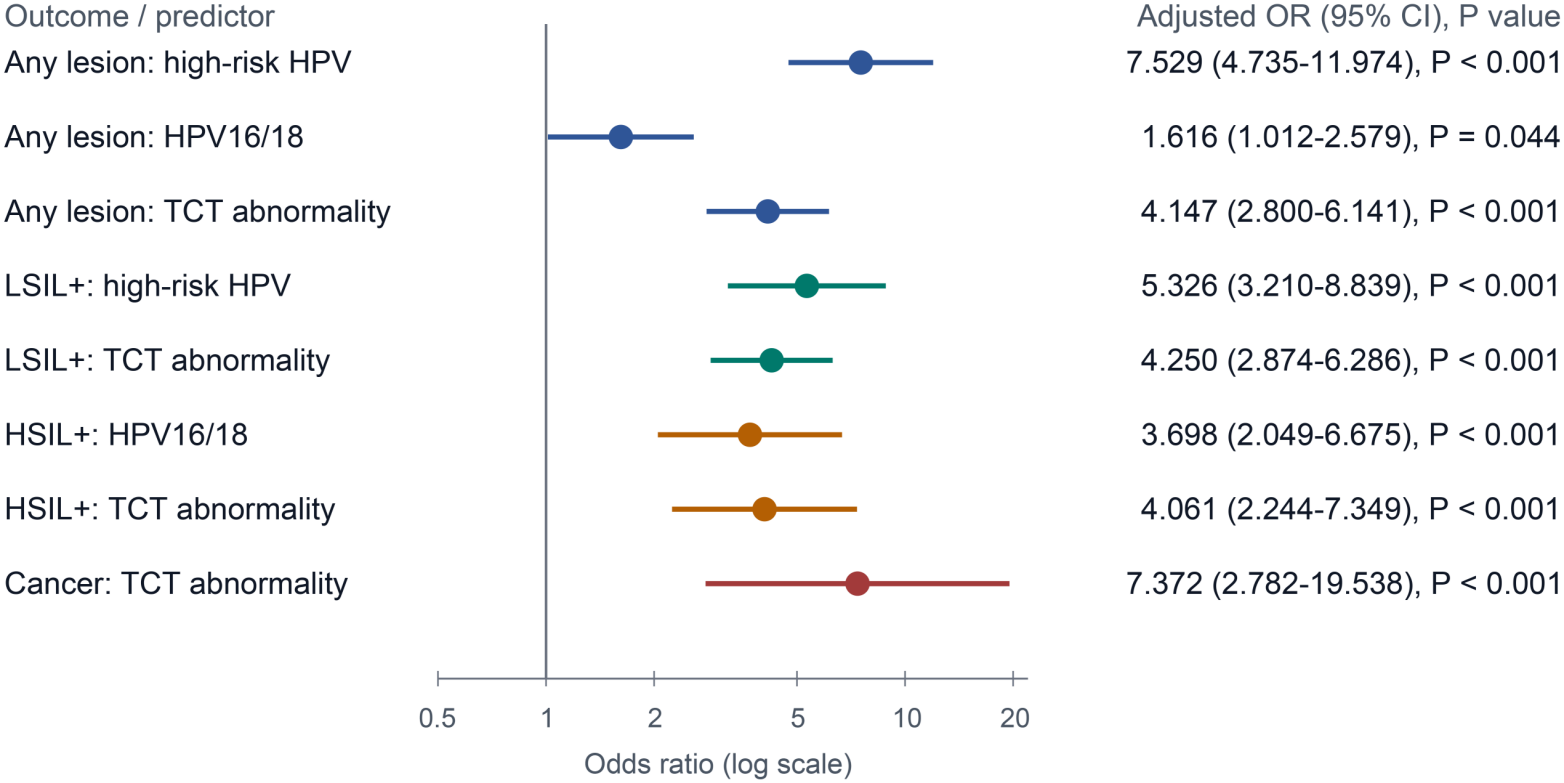
Predictors of histopathologic cervical lesions. Forest plot showing adjusted associations of BV, high-risk HPV positivity, HPV16/18 positivity, HPV multiple infection, and TCT abnormality with histopathologic outcomes in the cervical histopathology cohort (n=825).

In contrast, established HPV-related markers were strongly associated with histologic lesions. High-risk HPV positivity, HPV16/18 positivity, and TCT abnormality were independently associated with LSIL or worse and/or HSIL or worse, while TCT abnormality showed the strongest association with cancer (**Table 4** and **Figure 5**). In the HSIL-or-worse model, HPV multiple infection showed an inverse association after simultaneous adjustment for high-risk HPV and HPV16/18 positivity. Because HPV genotype variables are biologically overlapping, we performed additional collinearity and alternative-model analyses. VIFs in the full histopathology model were all below 2.0, suggesting no severe statistical collinearity. However, the inverse association for HPV multiple infection was attenuated and became non-significant when HPV multiple infection was modeled without simultaneous HR-HPV or HPV16/18 adjustment (aOR 0.717; 95% CI 0.388-1.325; P = 0.288), supporting a model-dependent interpretation rather than a protective biological effect (**Supplementary Tables S1**–**S3**).

**Table 4:** Multivariable associations with histologic lesions.

Outcome	Predictor	Adjusted OR (95% CI)	P value
Any target lesion	BV morphology positive	0.751 (0.432-1.306)	0.310
	High-risk HPV positivity	7.529 (4.735-11.974)	<0.001
	HPV16/18 positivity	1.616 (1.012-2.579)	0.044
	HPV multiple infection	1.009 (0.652-1.561)	0.969
	TCT abnormality	4.147 (2.800-6.141)	<0.001
LSIL or worse	BV morphology positive	0.576 (0.323-1.024)	0.060
	High-risk HPV positivity	5.326 (3.210-8.839)	<0.001
	HPV16/18 positivity	1.662 (1.066-2.590)	0.025
	HPV multiple infection	1.091 (0.715-1.664)	0.687
	TCT abnormality	4.250 (2.874-6.286)	<0.001
HSIL or worse	BV morphology positive	0.597 (0.244-1.463)	0.260
	High-risk HPV positivity	2.415 (1.135-5.138)	0.022
	HPV16/18 positivity	3.698 (2.049-6.675)	<0.001
	HPV multiple infection	0.473 (0.251-0.894)	0.021
	TCT abnormality	4.061 (2.244-7.349)	<0.001
Cancer	BV morphology positive	0.433 (0.095-1.979)	0.281
	High-risk HPV positivity	2.653 (0.840-8.378)	0.096
	TCT abnormality	7.372 (2.782-19.538)	<0.001

BV, bacterial vaginosis; HPV, human papillomavirus; TCT, thin-prep cytology test; LSIL, low-grade squamous intraepithelial lesion; HSIL, high-grade squamous intraepithelial lesion; OR, odds ratio; CI, confidence interval. The histopathology cohort was clinically selected and should not be interpreted as a randomly sampled subset of the full screening cohort. For the cancer outcome (n=38 events), only three predictors were included to avoid model overfitting. HPV16/18 positivity and HPV multiple infection were excluded from the cancer model because of the limited number of cancer events.

## 4 Discussion

In this two-year real-world pathology-microbiology study integrating vaginal fluorescence microscopy, HPV genotyping, cervical cytology, and histopathology, morphology-defined BV was associated with multiple HPV-related laboratory and screening abnormalities. BV-positive patients had higher odds of overall HPV positivity, high-risk HPV positivity, HPV multiple infection, TCT abnormality, and concurrent high-risk HPV positivity with TCT abnormality. These associations remained robust after adjustment for year, age, and vaginal microecological factors and were confirmed in a 90-day sensitivity analysis. However, BV was not independently associated with biopsy-confirmed histologic cervical lesions. Instead, high-risk HPV, HPV16/18, and TCT abnormality were the dominant predictors of histologic disease.

The present findings are broadly consistent with previous evidence linking BV or vaginal dysbiosis with HPV infection. A meta-analysis restricted to studies using Nugent criteria and PCR-based HPV detection reported a larger pooled association (OR 2.68) (Martins et al., 2022). A broader systematic review and meta-analysis of vaginal dysbiosis and a cross-sectional analysis of national surveillance data further supported positive associations between BV and HPV acquisition, persistence, and cervical disease outcomes (Brusselaers et al., 2019; Qi et al., 2024). The aOR of 1.611 for high-risk HPV positivity observed in our study is broadly consistent with the estimate reported by Gillet et al. (2011) and lower than that reported by Martins et al. (2022), which may reflect differences in BV diagnostic criteria, study populations, HPV detection methods, and adjustment strategies.

A notable finding was the strong association between morphology-defined BV and HPV multiple infection. The adjusted odds of HPV multiple infection were nearly two-fold higher among BV-positive patients, and this association was the strongest among the HPV-related outcomes. Multiple HPV infection may reflect increased susceptibility to viral acquisition, impaired local viral clearance, or repeated exposure in a cervicovaginal microenvironment with reduced Lactobacillus dominance and increased anaerobic bacterial burden (Cui et al., 2025; Gilbert et al., 2025). Although the present study could not evaluate HPV persistence or clearance, the observed association between morphology-defined BV and multiple HPV infection supports the hypothesis that vaginal dysbiosis may be correlated with more complex HPV infection patterns, although residual behavioral and clinical confounding cannot be excluded (Sheng et al., 2025).

Several biological mechanisms may explain the association between BV and HPV infection. BV is characterized by reduced Lactobacillus dominance, enrichment of anaerobic bacteria, increased vaginal pH, epithelial barrier disruption, and mucosal inflammation (Norenhag et al., 2020; Zukiene et al., 2025). These changes may facilitate HPV acquisition by weakening epithelial integrity, altering cervicovaginal mucus, and modifying local immune responses (Gilbert et al., 2025). BV-associated bacteria may also produce enzymes and metabolites that affect epithelial junctions and inflammatory signaling (Swidsinski et al., 2023). These mechanisms are compatible with the observed associations between BV, high-risk HPV positivity, and HPV multiple infection. Because the present study used routine morphologic BV assessment rather than microbial sequencing, these mechanisms remain biologically plausible explanations rather than directly measured pathways.

The attenuation of the BV–TCT association after additional adjustment for high-risk HPV is informative but should not be interpreted as formal mediation analysis. In the main model, BV was associated with TCT abnormality. However, after high-risk HPV positivity was added to the model, the association between BV and cytologic abnormality was substantially attenuated and no longer statistically significant. This suggests that HPV infection may partly account for the relationship between BV and cytologic abnormality (Schellekens et al., 2025). Alternatively, BV and HPV may share behavioral, immunologic, or local ecological determinants that cannot be fully separated in a retrospective design.

The histopathology findings provide an important downstream clinical context. BV was associated with HPV-related screening abnormalities in the full screening cohort, but this association did not translate into a measurable increase in LSIL+, HSIL+, or cancer among patients who underwent histopathologic evaluation. Previous meta-analyses have examined whether BV-related dysbiosis extends to CIN risk, but findings vary by BV definition, lesion threshold, and study design (Mortaki et al., 2020). In our study, high-risk HPV, HPV16/18, and TCT abnormality remained the principal predictors of histologic cervical lesions, supporting the central role of HPV genotype and cytology in identifying clinically significant disease. Recent Chinese cross-sectional evidence has also shown that HPV prevalence and genotype distribution differ among women with cervical or vaginal lesions, further supporting the importance of local population-specific data when interpreting HPV-related screening and histopathologic findings (Han et al., 2025).

The inverse association between HPV multiple infection and HSIL or worse in the fully adjusted histopathology model should not be interpreted as evidence of a protective biological effect. Although VIFs were below conventional thresholds for severe collinearity, high-risk HPV positivity, HPV16/18 positivity, and HPV multiple infection are biologically overlapping genotype variables. In alternative models, the inverse association between HPV multiple infection and HSIL or worse disappeared when HPV multiple infection was modeled without simultaneous adjustment for HR-HPV and HPV16/18. This finding is therefore best viewed as a model-dependent statistical pattern rather than a clinically actionable protective association.

The histopathology analysis requires especially careful interpretation. The 825-patient pathology subgroup was not a random sample of the full screening population; it was enriched for HPV positivity and TCT abnormality, consistent with referral for colposcopy, biopsy, or surgical pathology. This selection process may attenuate or distort the estimated association between BV and histologic lesions because referral was likely driven primarily by HPV and cytology rather than by BV status. Therefore, the null association between BV and histologic endpoints should be interpreted as a finding within a selected biopsy population, not as proof that vaginal microecology is unrelated to cervical carcinogenesis.

The year-to-year difference in BV positivity deserves caution. BV positivity declined from 15.95% in 2024 to 9.81% in 2025, while HPV and TCT abnormality rates remained similar. This pattern may reflect a true shift in patients undergoing vaginal fluorescence microscopy, but it may also reflect differences in specimen collection, interpretation thresholds, laboratory workflow, or reader composition. Because the dataset did not include operator-level quality indicators, we cannot determine the exact reason for this difference. We therefore adjusted for year, reported year-specific descriptive results, and recommend that future studies incorporate laboratory quality-control variables and interobserver agreement assessment.

This study has several strengths. The analysis was based on a large two-year real-world dataset and used patient-level deduplication across years, which reduced potential bias from repeated testing. HPV genotyping, TCT cytology, vaginal fluorescence microscopy, and pathology records were integrated using prespecified temporal matching windows, and the main findings were supported by a 90-day sensitivity analysis. By evaluating overall HPV positivity, high-risk HPV positivity, HPV multiple infection, cytologic abnormality, and histologic endpoints within the same clinical framework, the study provides real-world evidence on the relationship between vaginal microecological morphology and HPV-related clinical phenotypes.

Several limitations should be acknowledged. Because this was a retrospective observational study, causal inference cannot be established. BV was defined by vaginal fluorescence morphology rather than Nugent scoring, Amsel criteria, or molecular microbiome profiling. Although this morphology-defined approach reflects routine clinical practice and is scalable, it may not capture the full microbial community structure. HPV testing was based on genotyping rather than quantitative viral-load measurement, and the study could not evaluate HPV persistence, viral clearance, or time-dependent changes in genotype status. Residual confounding may substantially influence the reported adjusted odds ratios, particularly for HPV positivity and HPV multiple infection, because information on sexual behavior, number of sexual partners, condom use, smoking, contraceptive use, socioeconomic status, HPV vaccination, prior HPV infection, prior cervical procedures or treatment, and recent antibiotic use was unavailable. Therefore, the observed associations should not be interpreted as causal evidence that morphology-defined BV increases HPV risk.

Additional limitations include the significant year-to-year difference in BV positivity, which raises the possibility of unmeasured changes in the tested clinical population, specimen collection, slide interpretation, or laboratory workflow. No intentional change in the laboratory workflow, microscope system, reporting framework, or morphology-based diagnostic criteria was identified during the study period. All operators were trained before performing the assay, and reagent lots were accepted only after routine pre-use quality verification. Nevertheless, operator-level performance metrics and lot-specific quantitative quality-control indicators were unavailable, so residual measurement bias cannot be fully excluded. The histopathology cohort consisted only of patients who underwent biopsy or surgical pathology, representing a clinically selected subgroup rather than the full screening population. Multiple endpoints were evaluated, which may increase the possibility of false-positive findings. Cancer-specific analyses were limited by the small number of cancer cases and should be interpreted descriptively. Finally, the cross-sectional nature of the matched data limited assessment of temporal relationships between BV, HPV acquisition, HPV persistence, and lesion progression.

Despite these limitations, the findings have practical implications. Morphology-defined BV may identify women with a higher likelihood of HPV positivity, high-risk HPV infection, and multiple HPV infection in routine cervical screening. However, BV alone should not be considered a substitute marker for histologic cervical disease. Risk stratification for clinically significant lesions should continue to rely primarily on high-risk HPV genotype, cytology, and histopathologic confirmation. Future prospective studies with longitudinal HPV follow-up, standardized BV diagnosis, laboratory quality-control metrics, and molecular microbiome characterization are needed to clarify whether BV treatment or restoration of a Lactobacillus-dominant vaginal microbiota can improve HPV clearance or reduce HPV-related screening abnormalities (Zeng et al., 2023; Susetiati et al., 2025).

## 5 Conclusion

Morphology-defined BV was associated with HPV infection, high-risk HPV infection, HPV multiple infection, cytologic abnormality, and concurrent high-risk HPV positivity with cytologic abnormality in a two-year real-world pathology-microbiology study. However, BV was not independently associated with histologic cervical lesions among patients undergoing biopsy. These results suggest that routine morphologic evidence of BV is linked primarily to HPV-related infection and screening-positive phenotypes rather than to established histologic cervical disease. Prospective longitudinal studies using standardized BV diagnostic criteria and microbiome-based assessment are required before morphology-defined BV can be used for lesion-level risk prediction.

## Reporting checklist

A completed STROBE checklist is provided as **Supplementary Material**.

## Data Availability

The original contributions presented in the study are included in the article/**Supplementary Material**. Further inquiries can be directed to the corresponding authors.
