# Supplementary material for: Morphology-defined bacterial vaginosis and HPV-related cervical screening abnormalities: a two-year real-world study with histopathologic correlation
Source: Front Cell Infect Microbiol. 2026 Jul 22;16:1890650. doi: 10.3389/fcimb.2026.1890650 (PMC13437351; doi:10.3389/fcimb.2026.1890650)
Supplement: Supplementary file 1 [file Table1.docx]

STROBE Statement—checklist of items that should be included in reports of observational studies

|  | Item No | Recommendation | **Reported on page No.** |
| --- | --- | --- | --- |
| **Title and abstract** | 1 | (*a*) Indicate the study’s design with a commonly used term in the title or the abstract | Title (“real-world study”); Abstract §Methods |
|  |  | (*b*) Provide in the abstract an informative and balanced summary of what was done and what was found | Abstract (Background / Methods / Results / Conclusion) |
| Introduction | | |  |
| Background/rationale | 2 | Explain the scientific background and rationale for the investigation being reported | Introduction §1 (paras 1-3) |
| Objectives | 3 | State specific objectives, including any prespecified hypotheses | Introduction §1 (final para) |
| Methods | | |  |
| Study design | 4 | Present key elements of study design early in the paper | Methods §2.1 (retrospective real-world observational study) |
| Setting | 5 | Describe the setting, locations, and relevant dates, including periods of recruitment, exposure, follow-up, and data collection | Methods §2.1 (First Hospital of Changsha; Jan 2024-Dec 2025) |
| Participants | 6 | (*a*) *Cohort study*—Give the eligibility criteria, and the sources and methods of selection of participants. Describe methods of follow-up  *Case-control study*—Give the eligibility criteria, and the sources and methods of case ascertainment and control selection. Give the rationale for the choice of cases and controls  *Cross-sectional study*—Give the eligibility criteria, and the sources and methods of selection of participants | Methods §2.2 (inclusion and exclusion criteria) |
|  |  | (*b*) *Cohort study*—For matched studies, give matching criteria and number of exposed and unexposed  *Case-control study*—For matched studies, give matching criteria and the number of controls per case | N/A. Records were temporally linked by prespecified matching windows, but no exposed-unexposed or case-control matching design was used. |
| Variables | 7 | Clearly define all outcomes, exposures, predictors, potential confounders, and effect modifiers. Give diagnostic criteria, if applicable | Methods §2.4 (BV); §2.5 (HPV); §2.6-2.7 (TCT, histopathology); §2.9 (covariates) |
| Data sources/ measurement | 8* | For each variable of interest, give sources of data and details of methods of assessment (measurement). Describe comparability of assessment methods if there is more than one group | Methods §2.3 (specimen collection); §2.4 (fluorescence microscopy); §2.5 (HPV kit); §2.6-2.7 (TCT/histopathology) |
| Bias | 9 | Describe any efforts to address potential sources of bias | Methods §2.8 and §2.9; Discussion §4 (patient-level deduplication; prespecified 30-day matching window; year adjustment; clinically selected histopathology subgroup; residual confounding and measurement-bias limitations) |
| Study size | 10 | Explain how the study size was arrived at | Methods §2.2 (consecutive sampling; all eligible patients included) |
| Quantitative variables | 11 | Explain how quantitative variables were handled in the analyses. If applicable, describe which groupings were chosen and why | Methods §2.9 (continuous: median/IQR; categorical: counts/proportions) |
| Statistical methods | 12 | (*a*) Describe all statistical methods, including those used to control for confounding | Methods §2.9 (multivariable logistic regression; adjusted for year, age, microecological covariates) |
|  |  | (*b*) Describe any methods used to examine subgroups and interactions | No formal interaction analyses were performed. The histopathology analysis was conducted as a downstream subgroup/correlation analysis among patients with available cervical pathology records (n=825). |
|  |  | (*c*) Explain how missing data were addressed | Methods §2.9 (complete-case analysis; patients with incomplete data excluded per §2.2) |
|  |  | (*d*) *Cohort study*—If applicable, explain how loss to follow-up was addressed  *Case-control study*—If applicable, explain how matching of cases and controls was addressed  *Cross-sectional study*—If applicable, describe analytical methods taking account of sampling strategy | N/A (no complex sampling strategy; consecutive clinical records) |
|  |  | (*e*) Describe any sensitivity analyses | Methods §2.9 (90-day temporal-window sensitivity analysis; VIF collinearity assessment and alternative histopathology models) |

Continued on next page

| Results | | |  |
| --- | --- | --- | --- |
| Participants | 13* | (a) Report numbers of individuals at each stage of study—eg numbers potentially eligible, examined for eligibility, confirmed eligible, included in the study, completing follow-up, and analysed | Results §3.1; Fig. 1 (source records -> matched -> deduplicated -> analysed) |
|  |  | (b) Give reasons for non-participation at each stage | Methods §2.2 (exclusion criteria); Results §3.1; Fig. 1 |
|  |  | (c) Consider use of a flow diagram | Fig. 1 |
| Descriptive data | 14* | (a) Give characteristics of study participants (eg demographic, clinical, social) and information on exposures and potential confounders | Table 1 (baseline characteristics stratified by BV status) |
|  |  | (b) Indicate number of participants with missing data for each variable of interest | Methods §2.2 and §2.9 (patients with incomplete essential clinical information were excluded; missing or unavailable routine clinical variables were not imputed) |
|  |  | (c) *Cohort study*—Summarise follow-up time (eg, average and total amount) | N/A. This was a retrospective matched-record observational analysis rather than a longitudinal cohort study; no follow-up time was analysed. |
| Outcome data | 15* | *Cohort study*—Report numbers of outcome events or summary measures over time | N/A |
|  |  | *Case-control study—*Report numbers in each exposure category, or summary measures of exposure | N/A |
|  |  | *Cross-sectional study—*Report numbers of outcome events or summary measures | Results §3.1-3.5; Tables 1-4 (HPV, TCT, histopathology outcome rates reported) |
| Main results | 16 | (*a*) Give unadjusted estimates and, if applicable, confounder-adjusted estimates and their precision (eg, 95% confidence interval). Make clear which confounders were adjusted for and why they were included | Tables 2-4; Results §3.2-3.5 (univariable and multivariable ORs with 95% CI; confounders stated) |
|  |  | (*b*) Report category boundaries when continuous variables were categorized | N/A (no continuous variable categorized) |
|  |  | (*c*) If relevant, consider translating estimates of relative risk into absolute risk for a meaningful time period | N/A |
| Other analyses | 17 | Report other analyses done—eg analyses of subgroups and interactions, and sensitivity analyses | Results §3.4 (90-day temporal-window sensitivity analysis); Results §3.5 and Supplementary Tables S1-S3 (VIF collinearity assessment and alternative histopathology models) |
| Discussion | | |  |
| Key results | 18 | Summarise key results with reference to study objectives | Discussion §4 (first two paragraphs) |
| Limitations | 19 | Discuss limitations of the study, taking into account sources of potential bias or imprecision. Discuss both direction and magnitude of any potential bias | Discussion §4 (year-to-year BV difference; selection bias; single-centre; cross-sectional design) |
| Interpretation | 20 | Give a cautious overall interpretation of results considering objectives, limitations, multiplicity of analyses, results from similar studies, and other relevant evidence | Discussion §4 (final interpretation paragraphs) |
| Generalisability | 21 | Discuss the generalisability (external validity) of the study results | Discussion §4 (single tertiary-centre limitation; Chinese clinical population noted) |
| Other information | | |  |
| Funding | 22 | Give the source of funding and the role of the funders for the present study and, if applicable, for the original study on which the present article is based | Funding section (grant numbers listed; no role in study design or analysis) |

*Give information separately for cases and controls in case-control studies and, if applicable, for exposed and unexposed groups in cohort and cross-sectional studies.

**Note:** An Explanation and Elaboration article discusses each checklist item and gives methodological background and published examples of transparent reporting. The STROBE checklist is best used in conjunction with this article (freely available on the Web sites of PLoS Medicine at http://www.plosmedicine.org/, Annals of Internal Medicine at http://www.annals.org/, and Epidemiology at http://www.epidem.com/). Information on the STROBE Initiative is available at www.strobe-statement.org.
