# Supplementary material for: Morphology-defined bacterial vaginosis and HPV-related cervical screening abnormalities: a two-year real-world study with histopathologic correlation
Source: Front Cell Infect Microbiol. 2026 Jul 22;16:1890650. doi: 10.3389/fcimb.2026.1890650 (PMC13437351; doi:10.3389/fcimb.2026.1890650)
Supplement: Supplementary file 2 [file Table2.docx]

Supplementary Tables

**Supplementary Table S1.** Collinearity assessment for the full histopathology model

| Variable | R-squared | VIF | Interpretation |
| --- | --- | --- | --- |
| Pathology source year 2025 vs 2024 | 0.016 | 1.016 | No serious collinearity |
| Age, per 1-year increase | 0.053 | 1.055 |  |
| Morphology-defined BV | 0.021 | 1.021 |  |
| VVC morphology positive | 0.007 | 1.007 |  |
| HR-HPV positivity | 0.493 | 1.972 |  |
| HPV16/18 positivity | 0.203 | 1.255 |  |
| HPV multiple infection | 0.273 | 1.375 |  |
| TCT abnormality (ASC-US+) | 0.304 | 1.437 |  |

Abbreviations: BV, bacterial vaginosis; HPV, human papillomavirus; HR-HPV, high-risk human papillomavirus; TCT, thin-prep cytology test; VIF, variance inflation factor; VVC, vulvovaginal candidiasis.

**Supplementary Table S2.** Alternative logistic regression models for morphology-defined BV and histopathologic outcomes

| Histopathologic outcome | Model | Adjusted OR for morphology-defined BV | 95% CI | P value |
| --- | --- | --- | --- | --- |
| Any target lesion | Full overlapping HPV model | 0.751 | 0.432-1.306 | 0.310 |
|  | HR-HPV only model | 0.788 | 0.457-1.359 | 0.392 |
|  | HPV16/18 only model | 0.801 | 0.470-1.366 | 0.416 |
|  | HPV multiple infection only model | 0.796 | 0.464-1.367 | 0.408 |
|  | TCT only model | 0.933 | 0.557-1.562 | 0.792 |
|  | HPV markers without TCT model | 0.778 | 0.459-1.317 | 0.350 |
| LSIL or worse | Full overlapping HPV model | 0.576 | 0.323-1.024 | 0.060 |
|  | HR-HPV only model | 0.619 | 0.352-1.088 | 0.095 |
|  | HPV16/18 only model | 0.610 | 0.345-1.080 | 0.090 |
|  | HPV multiple infection only model | 0.596 | 0.334-1.062 | 0.079 |
|  | TCT only model | 0.704 | 0.404-1.226 | 0.215 |
|  | HPV markers without TCT model | 0.618 | 0.359-1.065 | 0.083 |
| HSIL or worse | Full overlapping HPV model | 0.597 | 0.244-1.463 | 0.260 |
|  | HR-HPV only model | 0.611 | 0.260-1.436 | 0.258 |
|  | HPV16/18 only model | 0.529 | 0.217-1.287 | 0.160 |
|  | HPV multiple infection only model | 0.695 | 0.294-1.643 | 0.407 |
|  | TCT only model | 0.651 | 0.278-1.525 | 0.323 |
|  | HPV markers without TCT model | 0.589 | 0.248-1.402 | 0.232 |
| Cancer | Full overlapping HPV model | 0.349 | 0.064-1.910 | 0.225 |
|  | HR-HPV only model | 0.433 | 0.095-1.979 | 0.281 |
|  | HPV16/18 only model | 0.288 | 0.057-1.459 | 0.133 |
|  | HPV multiple infection only model | 0.559 | 0.119-2.617 | 0.460 |
|  | TCT only model | 0.456 | 0.100-2.082 | 0.311 |
|  | HPV markers without TCT model | 0.359 | 0.072-1.780 | 0.210 |

Abbreviations: BV, bacterial vaginosis; CI, confidence interval; HPV, human papillomavirus; HR-HPV, high-risk human papillomavirus; OR, odds ratio; TCT, thin-prep cytology test; VVC, vulvovaginal candidiasis.

Note: All alternative histopathology models included pathology source year, age, morphology-defined BV, and VVC morphology positivity. HPV and TCT variables were then entered according to the model name. Morphology-defined BV was defined as the presence of clue cells or Gardnerella-like anaerobic bacteria on vaginal fluorescence microscopy.

**Supplementary Table S3.** Sensitivity analysis for HPV multiple infection and HSIL or worse

| Model | Variable | Adjusted OR | 95% CI | P value | Note |
| --- | --- | --- | --- | --- | --- |
| Full overlapping HPV model | HPV multiple infection | 0.473 | 0.251-0.894 | 0.021 | Modeled simultaneously with HR-HPV and HPV16/18; interpret cautiously |
| HPV multiple infection only model | HPV multiple infection | 0.717 | 0.388-1.325 | 0.288 | Modeled without simultaneous HR-HPV or HPV16/18 adjustment; inverse association disappeared |
| HPV markers without TCT model | HPV multiple infection | 0.475 | 0.257-0.876 | 0.017 | Modeled simultaneously with HR-HPV and HPV16/18; interpret cautiously |

Abbreviations: CI, confidence interval; HPV, human papillomavirus; HR-HPV, high-risk human papillomavirus; HSIL, high-grade squamous intraepithelial lesion; OR, odds ratio.

Note: The HPV multiple infection result in the full overlapping HPV model was treated as model-dependent and was not interpreted as a protective biological association.

**Supplementary Table S4.** Ninety-day sensitivity analysis.

| Outcome | Adjusted OR for BV (95% CI) | P value |
| --- | --- | --- |
| Overall HPV positivity | 1.566 (1.299-1.887) | <0.001 |
| High-risk HPV positivity | 1.619 (1.335-1.963) | <0.001 |
| HPV multiple infection | 1.966 (1.520-2.543) | <0.001 |
| TCT abnormality (ASC-US+) | 1.455 (1.119-1.890) | 0.005 |
| High-risk HPV positive + TCT abnormality | 1.551 (1.172-2.053) | 0.002 |

Adjusted odds ratios were estimated for morphology-defined BV using the 90-day HPV/TCT matching window. Models were adjusted for year, age, and vaginal microecological factors.
